# Multiple light-induced NO linkage isomers in the dinitrosyl complex [RuCl(NO)_2_(PPh_3_)_2_]BF_4_ unravelled by photocrystallographic and IR analysis

**DOI:** 10.1107/S2052252514023598

**Published:** 2015-01-01

**Authors:** Nicolas Casaretto, Sebastien Pillet, El Eulmi Bendeif, Dominik Schaniel, Anna K. E. Gallien, Peter Klüfers, Theo Woike

**Affiliations:** a Université de Lorraine, CRM2, UMR 7036, Vandoeuvre-les-Nancy, F-54506, France; b CNRS, CRM2, UMR 7036, Vandoeuvre-les-Nancy, F-54506, France; c Department Chemie, Ludwig-Maximilians-Universität, Butenandtstraße 5–13, 81377 Munich, Germany; d Institut für Struturphysik, TU Dresden, Zellescher Weg 16, Dresden, Germany

**Keywords:** crystal structure, light-induced isomerism, reversible isomerism, dinitrosyl compounds, photocrystallography, IR spectra

## Abstract

Multiple light-induced reversible metastable NO linkage isomers (PLIs) have been detected in the dinitrosyl compound [RuCl(NO)_2_(PPh_3_)_2_]BF_4_ by a combination of photocrystallographic and IR analysis. The IR signature of three PLI states has been clearly identified, with estimated populations of 59% (PLI-1), 8% (PLI-2) and 5% (PLI-3) for a total population of the metastable state of 72%. The structural configuration of the major component (PLI-1) has been derived by X-ray photocrystallography. In the ground state, the structure is characterized by a bent and a linear nitrosyl, the bent one being oriented towards the linear equatorial nitrosyl with an Ru—N—O angle of 133.88 (9)°. X-ray Fourier difference maps indicate a selectivity of the photo-isomerization process in PLI-1: only the bent NO ligand changes its position, while the linear NO is unaffected. After irradiation at 405 nm, the orientation is changed by rotation towards the Cl ligand opposite the linear NO, with an Ru—N—O angle in this new position of 109 (1)°. The photocrystallographic analysis provides evidence that, in the photo-induced metastable state, the bent NO group is attached to the Ru atom through the N atom (Ru—N—O), rather than in an isonitrosyl Ru—O—N binding mode. In the IR spectra, the asymmetric NO vibrational band shifts by −33 cm^−1^ to a lower value, whereas the symmetric band splits and shifts by 5 cm^−1^ to a higher value and by −8 cm^−1^ to a lower value. The down shift is a clear indication of the structural change, and the small upward shift in response to the new electronic configuration of the metastable structure. Variable-temperature IR kinetic measurements in the range 80–114 K show that the decay of the PLI-1 state follows an Arrhenius behaviour with an activation energy of 0.22 eV.

## Abstract 2

Photocrystallographic and IR analysis reveal light-induced reversible metastable NO linkage isomers in the dinitrosyl compound [RuCl(NO)_2_(PPh_3_)_2_]BF_4_.

## 1. Introduction   

Photo-induced metastable linkage NO isomers have been known for a long time in mono-nitrosyl transition metal complexes [*ML*_*x*_ *L*_*y*_(NO)] (Carducci *et al.*, 1997 ▶; Delley *et al.*, 1997 ▶; Gütlich *et al.*, 2001 ▶; Coppens *et al.*, 2002 ▶; Schaniel, Woike, Delley *et al.*, 2005 ▶; Bitterwolf, 2006 ▶). The central *M* atom is a transition metal (*e.g.* Mn, Fe, Ni, Co, Ru, Pt, Ir *etc.*), the ligands *L*_*x*_ and *L*_*y*_ are mono- or polydentate, and the complex can be anionic or cationic. Apart from a few exceptions, such as the half-sandwich compound [Ni(η^5^-Cp*)(NO)] (Fomitchev *et al.*, 1998 ▶), these complexes occur in octahedral form with a sixfold coordination of *M*. The NO ligand can adopt three binding modes: the linear (or bent) geometry of the ground state (GS) with a κ*N* binding mode, the metastable κ*O* isonitrosyl mode (MS1) and the metastable side-on κ^2^ *N*,*O* binding mode (MS2), as revealed by photocrystallographic techniques (Carducci *et al.*, 1997 ▶; Fomitchev & Coppens, 1996 ▶; Schaniel, Woike, Schefer & Petříček, 2005 ▶; Schaniel *et al.*, 2006 ▶). Using suitable wavelengths, a high population of up to nearly 100% of these metastable states can be induced in some compounds (Schaniel, Cormary *et al.*, 2007 ▶; Cormary *et al.*, 2009 ▶). The necessary condition for the generation of linkage isomers is the metal-to-ligand charge transfer (MLCT) transition from occupied metal *d* orbitals into empty antibonding π*(NO) orbitals, either by a direct *d*→π*(NO) transition, or by indirect transition as a relaxation from excited *d* orbitals into the π*(NO) orbital (Schaniel & Woike, 2009 ▶). After such an electronic excitation, the NO ligand undergoes a rotation by about 90° within 300 fs (Schaniel, Nicoul & Woike, 2010 ▶). This fast internal conversion from the excited GS potential into the MS2 potential is followed by a vibrational relaxation to the MS2 minimum, which occurs within about 10 ps (Gallé *et al.*, 2012 ▶). MS1 and MS2 are local minima on the potential energy surface of the ground electronic state, lying about 1 eV above the GS minimum, and they are separated from the GS by an energy barrier in the range 0.1–1.2 eV, depending on the ligands *L*_*x*_ and *L*_*y*_ and the central *M* atom. At sufficiently low temperatures the lifetime is nearly infinite, so many structural and spectroscopic techniques could be used to analyse all the changes produced by such metastable linkage isomers (Gütlich *et al.*, 2001 ▶).

Photo-induced linkage isomerism is not limited to nitrosyl complexes. A number of transition metal dinitrogen, nitro and sulfur dioxide complexes have been reported and structurally characterized by photocrystallographic techniques, revealing nitrito (κ*O*) and κ^2^ *S*,*O* linkage isomers (Kovalevsky *et al.*, 2002 ▶, 2005 ▶; Fomitchev *et al.*, 2000 ▶; Schaniel *et al.*, 2008 ▶; Schaniel, Mockus *et al.*, 2010 ▶; Brayshaw *et al.*, 2012 ▶; Hatcher *et al.*, 2014 ▶; Warren *et al.*, 2014 ▶). The discovery of photo-induced linkage NO isomers (PLIs) in the complex [Fe(CO)_2_(NO)_2_] (Klein *et al.*, 2010 ▶), which exhibits two NO ligands, raises the question of whether two different NO ligands on the same metal centre can form linkage isomers jointly or independently, enabling a combination of several metastable states for the dinitrosyl complex: NO1 (GS, MS1, MS2) and NO2 (GS, MS1, MS2). As shown by Gallien *et al.* (2014 ▶), diamagnetic penta-coordinated {*M*(NO)_2_}^8^ compounds (in the commonly used Enemark–Feltham notation for indicating electron counts; Enemark & Feltham, 1974 ▶) crystallize in two different structures: trigonal–bipyramidal (*TBPY*-5), with two linear NO ligands and an Ru(d^8^) electron configuration, and/or square-pyramidal (*vOC*-5), with a linear and a bent NO ligand and an Ru(d^6^) electron configuration. In terms of continuous shape measures (Alvarez *et al.*, 2005 ▶), the square-pyramidal structure is distorted towards a vacant octahedron, hence the *vOC*-5 label. In the *TBPY*-5 structure, the two NO ligands are bound in an identical linear configuration and the highest occupied molecular orbital (HOMO) is localized on the central metal atom as well as in equal parts on the NO ligands (Gallien *et al.*, 2014 ▶), leading to a highly degenerate situation in which the NO ligands are entangled. If, on the other hand, one of the NO ligands already exhibits a bent configuration in the GS, such as in the *vOC*-5 geometry, one preferred ligand may possibly isomerize while the other remains in the GS geometry, showing only a minor adjustment of bond lengths and angles resulting from the changed electronic configuration of the metal atom. It is conceivable that the electron density of the NO ligand in the bent *M*—N—O structure, which is formally negatively charged, is reduced by the HOMO–LUMO as a ligand-to-metal charge-transfer transition (LMCT; LUMO = lowest unoccupied molecular orbital) so that the angle increases up to about 180°, and in this unstable position the isomerization occurs. However, it is also possible that, in this unstable configuration, the linear NO ligand begins to rotate and the bent one remains stable as a linear NO. Such scenarios can be elucidated by photocrystallographic experiments.

**Figure 1:**
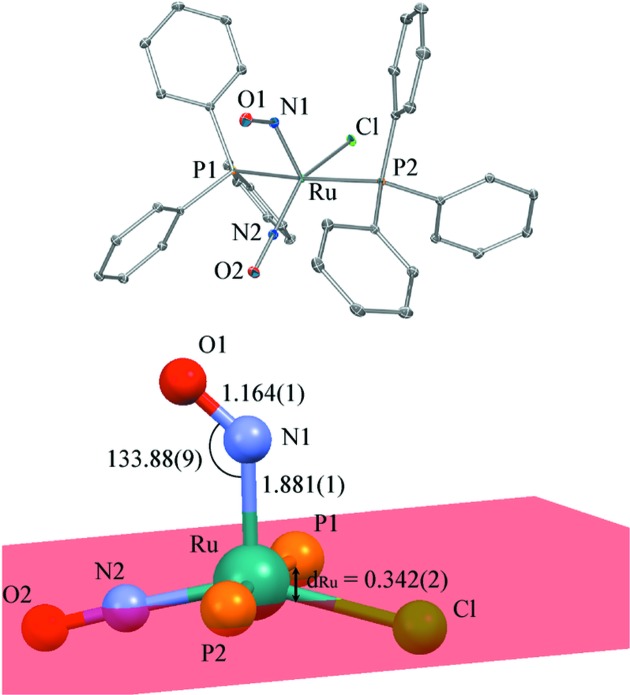
*ORTEP* (Burnett & Johnson, 1996 ▶) views of the ground state of [RuCl(NO)_2_(PPh_3_)_2_]BF_4_, with selected atomic labelling and relevant structural parameters (distances and angles in Å and °). The basal plane is depicted in red. Displacement ellipsoids are plotted at the 50% probability level.

In the IR spectra of the complexes [Fe(CO)_2_(NO)_2_] and [Ru*X*(NO)_2_(P*R*_3_)_2_]BF_4_ (Klein *et al.*, 2010 ▶; Gallien *et al.*, 2014 ▶) with *X* = Cl, Br or I, and P*R*_3_ = PPh_3_, PPh_2_Bn, PCy_3_, PCyp_3_ or P*^i^*Pr_3_, it was found that both stretching vibrations of the NO ligands are shifted upon light illumination, so that the force constants of both NO ligands are altered in the excited state. On its own, this observation does not allow definitive conclusions to be drawn about the structural configuration of the NO ligands. In order to investigate this phenomenon, and to answer the question of whether both NO ligands change their structural configuration, we studied the ruthenium complex [RuCl(NO)_2_(PPh_3_)_2_]BF_4_, using a combination of IR spectroscopy and X-ray photocrystallographic experiments. In this complex, one of the NO ligands is linear while the second NO is bent, in agreement with an overall *vOC*-5 structure (Fig. 1 ▶).

## 2. Experimental   

All compounds were synthesized according to literature procedures, as described by Gallien *et al.* (2014 ▶). Red crystals of [RuCl(NO)_2_(PPh_3_)_2_]BF_4_, suitable for X-ray crystallography, were formed on cooling the reaction solution to ambient temperature.

### 2.1. IR spectroscopy   

IR measurements were performed at *T* = 10 K, whereby the sample is kept in a vacuum inside a closed-cycle cryostat, using a Nicolet 5700 FT-IR spectrometer with a resolution of 2 cm^−1^. The sample was ground, mixed with KBr and pressed into pellets. The KBr pellets were bonded onto the cold finger of the cryostat using silver paste, and irradiated through KBr windows with laser light of wavelength 405 nm for population, and several lasers of wavelength in the range 660–1064 nm for depopulation. The thermal decay for the determination of the activation energy was determined from the decrease in the band area in steps of 2 K in the range 80–114 K, and by detecting the exponential decrease in the band area at a fixed temperature.

### 2.2. UV–vis absorption spectroscopy   

Absorption spectra were recorded on KBr pellets, such as those used for the IR spectroscopy, in the 350–800 nm range using a CARY 4000 spectrophotometer equipped with a closed-cycle cryostat. The sample was irradiated through quartz windows with laser light of wavelength 445 nm for population, and subsequently warmed to 50, 80 and 100 K for thermal depopulation.

### 2.3. Photocrystallographic data collection and structure refinement details   

Single-crystal X-ray diffraction data were collected on a SuperNova Microfocus diffractometer (Agilent, Oxfordshire, UK) equipped with a two-dimensional ATLAS detector, using Mo *K*α radiation (λ = 0.71073 Å) and a Helijet He open-flow cryosystem (Oxford Diffraction, Oxfordshire, UK). The temperature was fixed at 10 K. A single crystal was mounted on a glass fibre using vacuum grease. Diffraction data were first collected at 10 K in the ground state (GS) using ω scans. The unit-cell determination and data reduction were performed using the *CrysAlisPRO* program suite (Oxford Diffraction, 2011 ▶) on the full data set. 72 353 reflections were measured up to a maximum resolution of sin(θ)/λ = 0.77 Å^−1^ and merged to 13 009 unique reflections (*R*_int_ = 0.0408). A numerical absorption correction was performed. The corresponding structure was solved in the space group *P*2_1_/*c* by direct methods using the *SHELXS97* program (Sheldrick, 2008 ▶) and refined on *F* ^2^ by weighted full matrix least-squares methods using the *SHELXL97* program (Sheldrick, 2008 ▶). All non-H atoms were refined anisotropically. H atoms were located in difference Fourier maps and treated using a riding model, constraining the isotropic displacement parameters to 1.2*U*_eq_ of the parent C atom.

A fresh second sample was selected and irradiated at 10 K with a diode laser at 405 nm (*P* = 70 mW) for 40 min, until the photo-stationary state was reached. According to the UV–vis absorption spectra, this wavelength corresponds to an absorption band of the GS, which decreases in the photo-irradiated state (see supporting information). Two bands could indeed be found in the GS at 544 and at 405 nm. From the difference between the PLI and GS, two new bands are resolved at 603 and 482 nm (see Fig. S15 in the supporting information), indicating the spectral ranges favourable for population (centred at 405 nm) and depopulation (centred at 603 nm) of the PLI. Based on IR spectroscopy measurements (see above), a sufficiently high conversion ratio was expected with illumination at 405 nm. Complete diffraction data were collected in the photo-stationary state; no space-group change occurs with respect to the ground state (space group *P*2_1_/*c*). The unit-cell parameters change only slightly from *a* = 19.0673 (6), *b* = 9.8391 (2) and *c* = 20.5654 (6) Å in the GS to *a* = 19.111 (2), *b* = 9.8741 (6) and *c* = 20.588 (2) Å in the photo-induced state. The angle β decreases from 117.580 (4)° to 117.169 (14)°. The unit-cell volume therefore expands marginally by 1.1% [from 3419.7 (2) to 3456.4 (6) Å^3^]. 28 875 reflections were measured up to a maximum resolution of sin(θ)/λ = 0.69 Å^−1^, and merged to 8635 unique reflections (*R*_int_ = 0.0987). Empirical absorption correction was performed.

**Figure 2:**
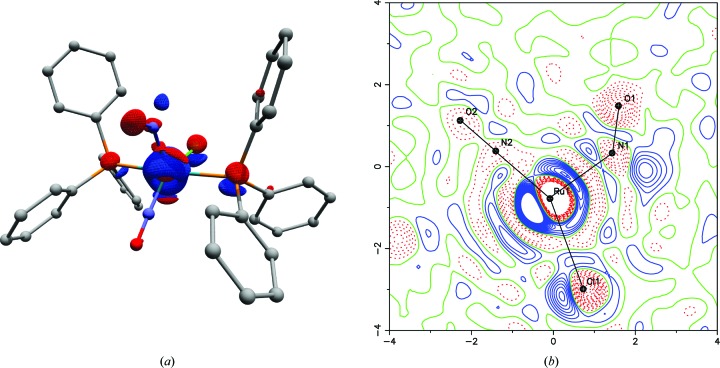
(*a*) A three-dimensional photo-difference map, with an isosurface of ±4.0 e Å^−3^ (red denotes negative and blue positive) at 10 K after irradiation at 405 nm. The map is based on 8634 independent measured reflections. (*b*) A section of the photo-difference map in the Ru/N1/N2 plane, with an isocontour of ±1.0 e Å^−3^ (red denotes negative and blue positive).

Photo-difference maps were calculated for visualization of the light-induced changes in electron density, and for identification of the related structural changes from the GS to the PLI (Fig. 2 ▶). Common independent reflections between the GS and photo-irradiated state were used to compute the experimental X-ray photo-difference map by Fourier transform of the difference [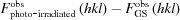], using the structure factor phases from the GS structural refinement. 8634 common independent reflections were included in the calculation, which comprises 89% of possible reflections up to a resolution of sin(θ)/λ = 0.69 Å^−1^. The Fourier maps are therefore reliable.

The calculated photo-difference maps revealed a pronounced structural reorganization around the bent nitrosyl ligand (see §3 for further discussion), with clear indications for the presence of predominantly two molecular species, GS and PLI-1 (see below for nomenclature). No evidence was found for any electron-density residues in the vicinity of the linear nitrosyl (as might be expected for an MS2 state of the linear nitrosyl). There was no indication for introducing other structural configurations into our model. In the present work, the population of PLI-1 could not be determined precisely by optical transmission experiments in the diffraction experimental conditions, so the respective populations *P* of the GS (*P*_GS_) and PLI-1 (*P*_PLI-1_) are necessary parameters of the structural refinement with the constraint *P*_GS_ + *P*_PLI-1_ = 1. We did not fix the population of the photo-irradiated state to the value derived from the IR analysis either, since a different photo-conversion might be expected owing to the different forms of the sample: a microcrystalline powder mixed with KBr in the IR spectroscopy, and a single crystal in the photocrystallography.

The photo-irradiated state structure was refined on *F* by weighted full matrix least-squares methods using the *JANA2006* program (Petříček *et al.*, 2006 ▶). Several refinement strategies, which are discussed in detail in the supporting information, were applied step by step in order to deconvolute the molecular structure of the GS and PLI-1; only the most relevant structural model is considered hereinafter, in which the entire GS species is considered as a rigid group (rigid group 1), with starting atomic positions taken from the GS molecular structure. The atomic displacement parameters were fixed at the GS structure values for all atoms except for O1 and N1 of the bent nitrosyl, which were refined anisotropically. For the PLI-1 species, the Ru atom and atoms O1 and N1 of the bent nitrosyl were freely refined anisotropically. The remaining atoms of the PLI-1 molecule were treated as a rigid group (rigid group 2) using the GS molecular structure. The atomic displacement parameters of the PLI-1 atoms N1 and O1 were constrained to have the same values as the refined ones of the GS atoms N1 and O1. The refined population of PLI-1 is 52 (1)%. Although other possible models gave better refinement statistics (see supporting information) at the expense of many more refined parameters, we consider that this highly constrained model is the most physically meaningful one. The structural degrees of freedom we have introduced, refining the position and displacement parameters of N1, O1 and Ru, allow us to take account of the reorganization in the photo-induced state of the bent nitrosyl and displacement of the Ru atom from the basal plane containing atoms N2, O2, P1, P2 and Cl.

Crystallographic details are provided in Table 1 ▶ and in the supporting information; selected bond lengths and angles are summarized in Table 2 ▶.

## 3. Results and discussion   

### 3.1. Structural signature of the photo-isomerization   

The crystal structure of [RuCl(NO)_2_(PPh_3_)_2_]BF_4_ consists of discrete five-coordinated [RuCl(NO)_2_(PPh_3_)_2_]^+^ cations and BF_4_^−^ counterions. The cationic complex (Fig. 1 ▶) may be described as a distorted square-planar pyramid, a vacant octahedron (*vOC*-5), in which two *trans* phosphane ligands, the Cl atom and one linear nitrosyl group make up the basal plane. The second nitrosyl group is located at the apex, with the Ru atom displaced by 0.342 (2) Å from the basal plane towards the apical nitrosyl (labelled *d*_Ru_ in Fig. 1 ▶). Although the crystal packing is very different, the molecular structure of the [RuCl(NO)_2_(PPh_3_)_2_]^+^ cation in the present compound is very similar to the PF_6_^−^ analogue reported previously (see Fig. S2 in the supporting information; Pierpont & Eisenberg, 1972 ▶). As seen for the PF_6_^−^ analogue, the apical nitrosyl group N1—O1 is coordinated in a bent manner, with a longer Ru—N1 distance of 1.881 (1) Å compared with the Ru—N2 distance of 1.762 (1) Å for the linear nitrosyl. The corresponding Ru—N—O angles are 133.88 (9) and 178.5 (1)° for the bent and linear conformations, respectively. The bent geometry and difference in bond length are attributed to a strong π back-bonding interaction of Ru with the linear nitrosyl present as NO^+^, while a much weaker π back-bonding interaction occurs for the bent nitrosyl NO^−^. This different ability originates from the formal +1 and −1 charges of the linear and bent nitrosyl, respectively. The sp^2^ hybridization of the N atom results in the close to 120° bent Ru—N—O geometry. Furthermore, the bent nitrosyl is oriented towards the linear equatorial N2—O2 group. This is common for such tetragonal pyramidal complexes, that the bent nitrosyl is oriented towards the ligand of greatest π-bonding ability, resulting from a weak donor–acceptor interaction between the lone pair of the apical nitrosyl O atom and the π*(NO) orbital of the linear nitrosyl (Pierpont & Eisenberg, 1972 ▶). This situation is similar in the PF_6_^−^ compound, and has also been encountered in other similar Ru–dinitrosyl complexes discussed by Gallien *et al.* (2014 ▶).

The crystal packing of [RuCl(NO)_2_(PPh_3_)_2_]BF_4_ is depicted in Fig. S1 in the supporting information. It consists of [RuCl(NO)_2_(PPh_3_)_2_]^+^ cations connected through a network of C—H⋯F, C—H⋯O and C—H⋯Cl hydrogen bonds and π−π interactions. In the ground state, neither of the nitrosyl ligands is involved in short hydrogen bonds. The shortest O1⋯H distances [O1⋯H36 = 2.734 (1), O1⋯H6 = 2.790 (1) and O1⋯H21 = 2.735 (1) Å] and O2⋯H distance [O2⋯H27 = 2.825 (1) Å] correspond to very weak hydrogen bonds.

The *ORTEPIII* (Burnett & Johnson, 1996 ▶) plot (Fig. 1 ▶) shows a difference between the atomic displacement parameters of the two nitrosyl ligands, characterized by slightly larger ellipsoids for the bent nitrosyl [*U*_iso_(N1) = 0.0078 (2), *U*_iso_(O1) = 0.0112 (2), *U*_iso_(N2) = 0.0070 (2) and *U*_iso_(O2) = 0.0110 (2) Å^2^].

X-ray diffraction measurements on the photo-excited sample enable the determination of the full three-dimensional structure of the PLI-1 configuration. Since photo-excitation in nitrosyl compounds is a local phenomenon, the overall structure (space group, packing, unit cell) does not change noticeably, and hereinafter we limit the discussion of photo-excitation structural reorganization to the cation and especially the Ru(NO)_2_ fragment. Complete structural information can be found in the supporting information.

Fig. 2 ▶ shows the three-dimensional experimental photodifference map after exciting the sample at 10 K with 405 nm light for 40 min. This map clearly highlights a slight displacement of the entire molecule in the PLI-1 state, as shown by electron-deficient regions (coloured red) centred on the position of the heaviest atoms in the ground state (*i.e.* Ru, P1, P2 and Cl), as well as adjacent electron-density accumulations (coloured blue). This means that too much electron density is subtracted by the 100% population of the GS in the red-coloured regions.

**Figure 3:**
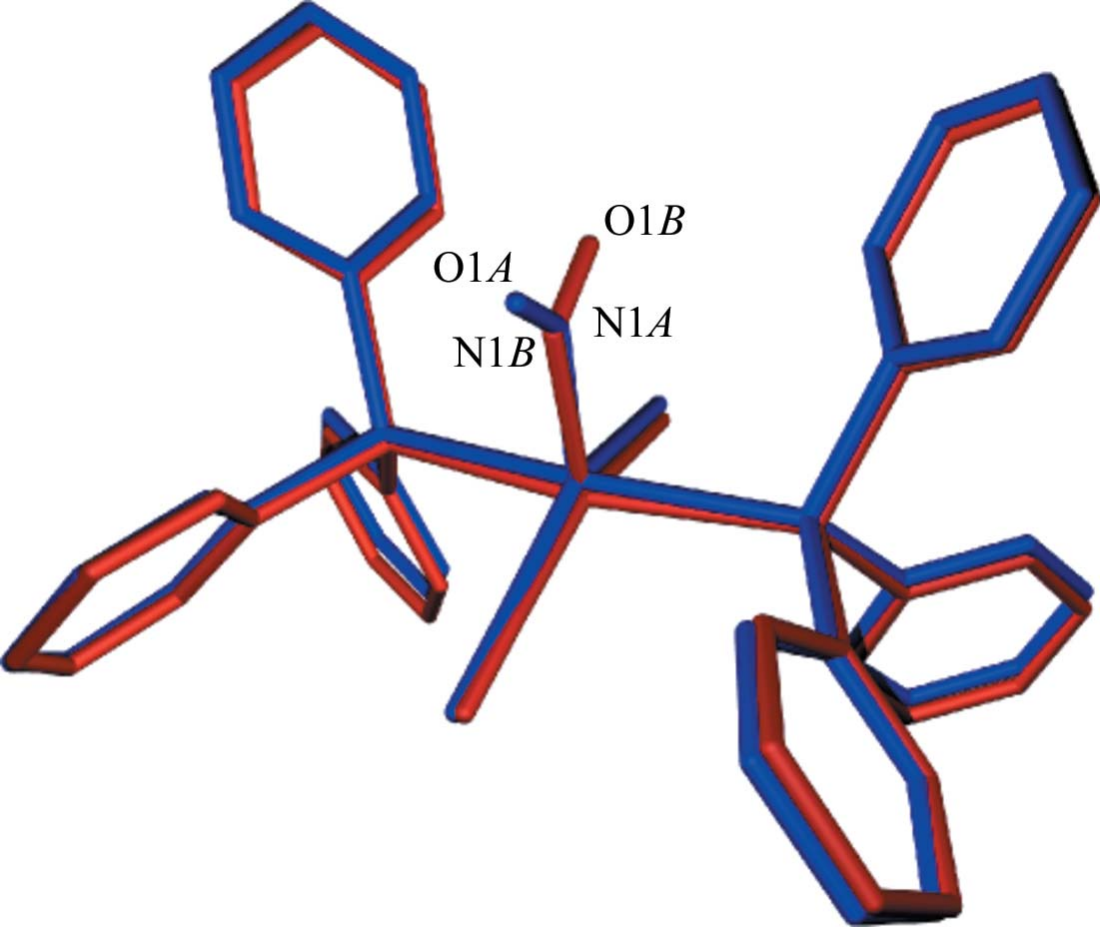
A structural model of the photo-irradiated state upon photo-excitation, corresponding to a superposition of the GS species (in blue and treated as a GS rigid group, with a refined population of 48%) and the PLI-1 species (in red, with a refined population of 52%, with atoms Ru, N1*B* and O1*B* freely refined and the remaining atoms treated as a rigid group).

In addition, strongly electron-deficient regions located at the positions of O1 and N1 in the GS bent NO1 nitrosyl, together with two positive regions, indicate an important structural reorganization. The two positive regions in the photodifference map would correspond to a nitrosyl in the PLI-1 state with a bent configuration, oriented towards the Cl atom rather than the linear nitrosyl. This was modelled using a superposition of two molecular entities, one corresponding to the unreacted GS and the other to the PLI-1 state. As such, two NO conformations were used (see Fig. 3 ▶), one corresponding to the GS conformation [Ru—N1*A*—O1*A* = 133.88°, Ru—N1*A* = 1.881 Å and N1*A*—O1*A* = 1.164 Å] with an occupancy factor much lower than unity [48 (1)%], and a second NO group adopting a bent configuration [Ru—N1*B*—O1*B* = 109 (1)°, Ru—N1*B* = 2.13 (1) Å and N1*B*—O1*B* = 1.06 (3) Å], with a refined population of 52 (1)%. This obtained population of PLI-1 is consistent with the very similar peak heights for the two positions of the O1 atom in the *F*_obs_ Fourier electron-density map (atoms O1*A* and O1*B* in Fig. S3; see supporting information). The bent configuration of the photo-induced state can be modelled by either an N-bound or an O-bound (isonitrosyl) NO ligand; the corresponding refinement agreement *R* values are equivalent and the residual electron-density maps do not allow for an unambiguous decision either. Inspection of the atomic displacement parameters of atoms N1*A* and O1*A* for the two hypotheses (*i.e.* Ru—N1*B*—O1*B* and Ru—O1*B*—N1*B*) may provide further indications of the structural configuration of the PLI-1. The refinement of the PLI-1 as a nitrosyl (Ru—N1*B*—O1*B* case) results in quite consistent atomic displacement parameters for both N1*B* [*U*_iso_(N1*B*) = 0.028 (4) Å^2^] and O1*B* [*U*_iso_(O1*B*) = 0.029 (4) Å^2^]. In contrast, refinement of the PLI-1 as an isonitrosyl (Ru—O1*B*—N1*B* case) leads to unreasonably high values for O1*B* [*U*_iso_(O1*B*) = 0.041 (4) Å^2^] and much lower values for N1*B* (*U*_iso_(N1*B*) = 0.018 (4) Å^2^]. This is an indication that the assignment of the atom connected to Ru (O1*B*) as oxygen (too much electron density) is clearly wrong, and must rather be taken as nitrogen. Refining a structure using such a split-atom model could lead to biased atomic displace­ment parameters if the atomic positions in the GS and metastable state are close to each other (Legrand *et al.*, 2007 ▶), but this is not the case here for the terminal atom of the NO group (whether it is attached to Ru through the N or O atom). The present structural analysis provides strong clues that, in the PLI-1 state elucidated by photocrystallography, the NO ligand corresponds to a nitrosyl binding mode, rather than an isonitrosyl as characterized in mononitrosyl transition metal compounds.

**Figure 4:**
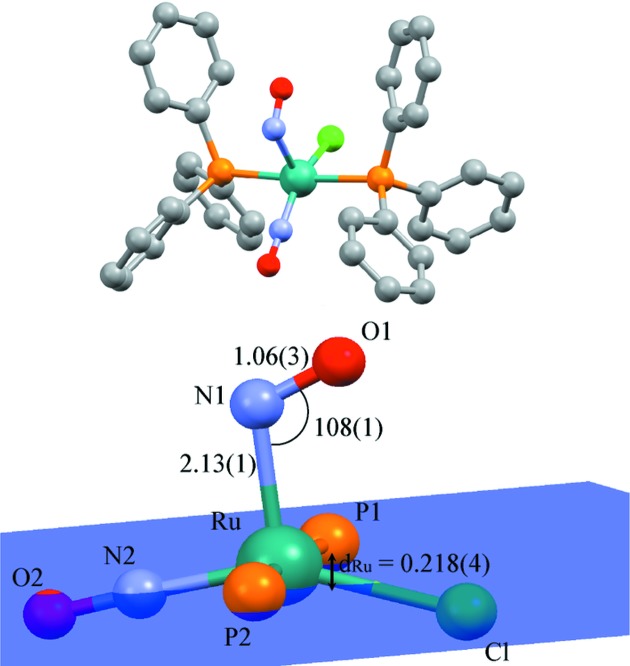
Structural models of the PLI-1 state upon photo-excitation. The basal plane is depicted in blue (distances and angles in Å and °).

As shown in Fig. 4 ▶, the major structural feature of the PLI-1 is a bent configuration, with an Ru—N1*B*—O1*B* angle much lower [109 (1)°] than the GS [133.88 (9)°]. In particular, the NO group is located in the direction of the Cl ligand instead of the linear NO ligand of the GS configuration. On the linear NO ligand, no significant changes to the structure could be detected after photo-excitation, as confirmed by the photo-difference map (Fig. 2 ▶). This indicates either that this ligand is not subject to PLI or that it exhibits a rather low population of an eventual isonitrosyl configuration, which is very hard to detect using X-ray diffraction (Carducci *et al.*, 1997 ▶; Schaniel *et al.*, 2006 ▶). As presented in Table 2 ▶, nearly all atomic distances and angles of the first Ru coordination are changed by the isomerization. As for the GS, the Ru atom is located in a distorted *vOC*-5, displaced by 0.218 (4) Å from the basal plane formed by the two P atoms, the Cl atom and the linear nitrosyl group; this is lower than the GS value. The Ru—Cl distance is shortened by 0.02 Å in the PLI-1, while the Ru—P distances remain almost constant and the Cl—Ru—N1 and Cl—Ru—N2 angles increase by 3.5° and 6.9°, respectively.

### 3.2. Influence of the crystal environment   

The change in conformation of the bent nitrosyl fragment is associated with the formation of two hydrogen bonds with O1⋯H distances of O1⋯H21 = 2.29 (1) and O1⋯H22 = 2.51 (1) Å, which are much shorter than the hydrogen bonds in the GS. These interactions are most probably at the origin of the stabilization of the orientation of the bent nitrosyl in the PLI-1 (see Fig. S9 in the supporting information).

**Figure 5:**
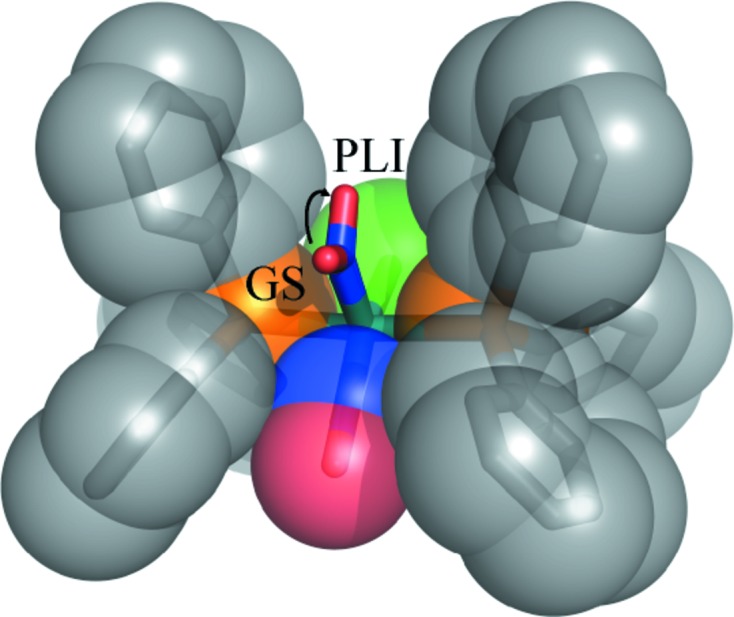
The van der Waals surface of the GS. The arrow indicates a possible trajectory of the photo-linkage isomerism.

The necessary conditions for the photogeneration of nitrosyl linkage isomers in [*ML*_5_NO] compounds has been reviewed by Schaniel & Woike (2009 ▶), discussing the fundamental electronic optically induced processes (*i.e.* MLCT or d→d transition) and the presence of a minimum in the excited-state potential between the GS and MS2 or MS1, both of these being of purely unimolecular origin. It has further been shown by theoretical density functional theory studies (Delley, 2008 ▶) that, although the global behaviour is retained, the crystal environment significantly influences the properties of nitrosyl compounds, especially the vibrational properties of the NO ligand (*i.e.* the shift of the NO stretching frequency on going from the GS to MS1 or MS2), as well as the configuration energy and transition state energy along the photo-linkage isomerism path on the energy hypersurface. The crystal environment may even sometimes hinder the observation of some configurations (Phillips *et al.*, 2010 ▶; Kawano *et al.*, 2000 ▶). Compared with liquid reactions, a steric factor may play a substantial role in solid-state isomerism. The van der Waals surface of the GS species, calculated using the GS structural configuration derived from the crystallographic analysis, is given in Fig. 5 ▶. It is obvious that steric effects render a solid-state isomerism possible by direct libration of the bent nitrosyl to the PLI-1 configuration in the plane defined by the Ru atom, the GS bent NO group and the PLI-1 bent NO group. A close inspection of the distribution of free cavity space within the crystal structure of the GS material may help in understanding the possible photo-isomerization. As is evident from Fig. S10 in the supporting information, the cavity spaces, defined as the region of the unit cell which could accommodate probe spheres of 0.8 Å radius not entering the van der Waals surfaces of neighbouring atoms, are located at only two positions, one of which is located close to the position of the bent NO group after photo-isomerization to the PLI-1 state. Accordingly, steric effects do not hinder the photo-isomerization, and a possible trajectory is depicted in Fig. 5 ▶.

### 3.3. Low-temperature IR spectroscopy   

**Figure 6:**
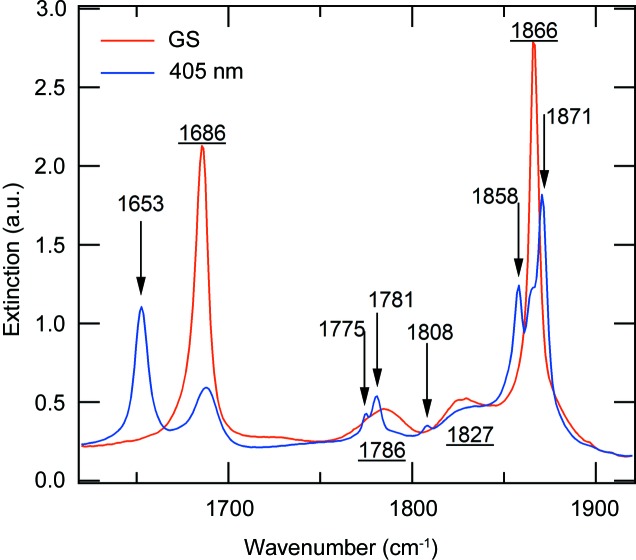
IR spectra (range 1620–1920 cm^−1^) of [RuCl(NO)_2_(PPh_3_)_2_]BF_4_ at 10 K. Arrows indicate new lines arising upon illumination with light of wavelength 405 nm. Underlined numbers refer to the positions of the GS bands.

To gain more insight, let us consider the results of IR spectroscopy on the PLI. Fig. 6 ▶ shows the IR spectra of the [RuCl(NO)_2_(PPh_3_)_2_]BF_4_ complex in the wavenumber range corresponding to NO vibrations, measured at 10 K in the ground state and after photo-excitation at 405 nm.

In the GS we find the two known (Gallien *et al.*, 2014 ▶) symmetrically and asymmetrically coupled NO stretching vibrations at 1866 and 1686 cm^−1^. We note in between two broad lines at 1786 and 1827 cm^−1^.

**Figure 7:**
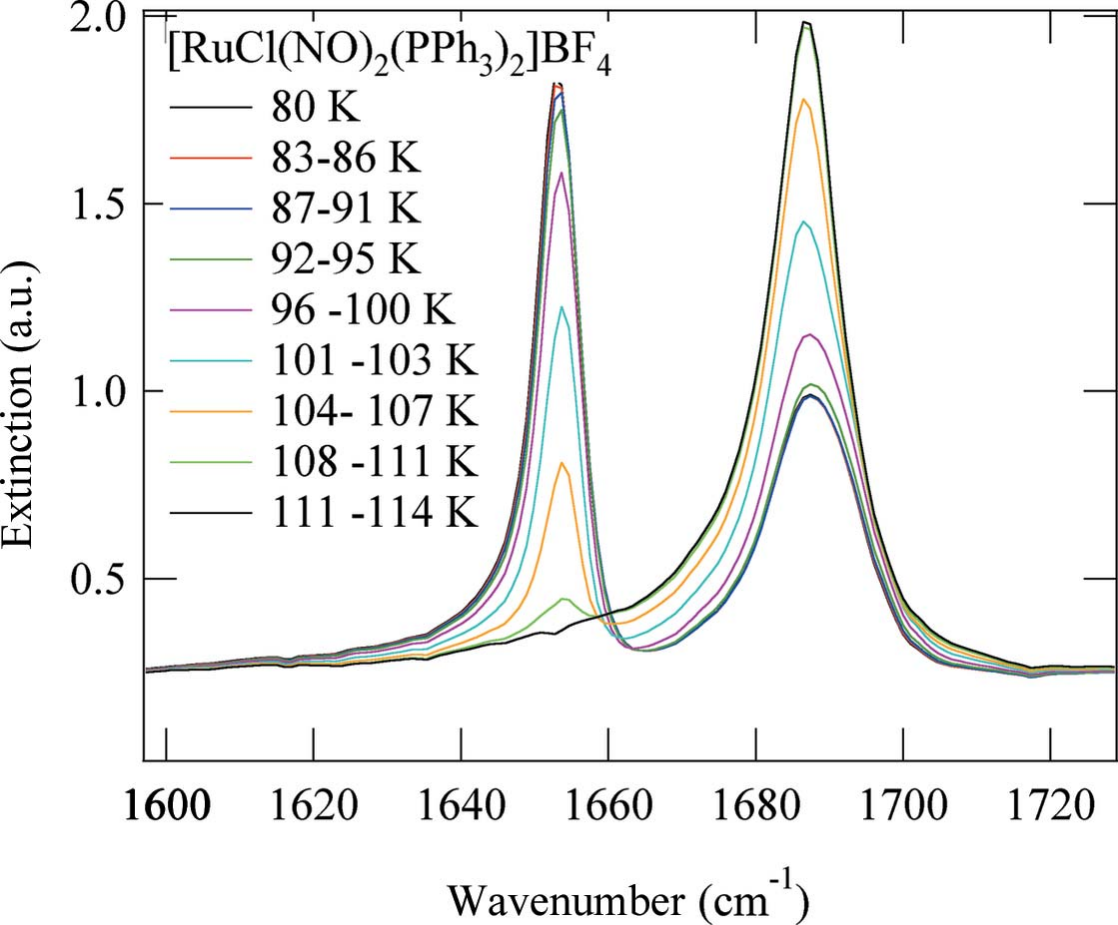
The decay of PLI-1 in the temperature range 80–114 K, with one isosbestic point. The measurement was performed dynamically while raising the temperature at a constant rate.

**Figure 8:**
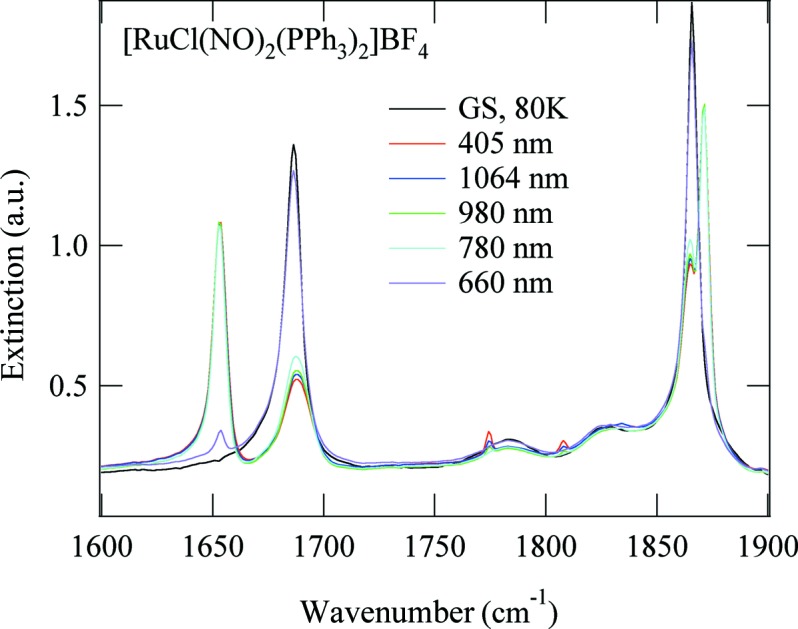
IR spectra of [RuCl(NO)_2_(PPh_3_)_2_]BF_4_ at 80 K in the GS (black line), after illumination at 405 nm to populate the PLIs (red line), and after further illumination at 660, 780, 980 and 1064 nm. Back-transfer to the GS is observed at 660 nm.

After photo-excitation at 405 nm we observe a decrease in the area of these four lines and the appearance of six new lines. From the decrease in the area of the GS band at 1686 cm^−1^ we infer a total population of about 72% of the PLIs. We will group the six photo-induced bands into three pairs for further discussion: 1871 and 1653 cm^−1^, 1858 and 1781 cm^−1^, and 1808 and 1775 cm^−1^. These three pairs show different behaviours as a function of temperature and illumination wavelength, so we consider them to be signatures for three different metastable linkage isomers. The one with the highest population identified *via* the two bands at 1871 and 1653 cm^−1^ corresponds to the PLI investigated by the X-ray diffraction experiment (PLI-1). It is thus a linkage isomer of the bent NO group, yielding a relatively large shift of −33 cm^−1^ for the asymmetric mode and a comparatively small shift of 5 cm^−1^ for the symmetric mode. These two bands disappear upon heating at a temperature of about 110 K (Fig. 7 ▶). Furthermore, these bands are not affected by irradiation with wavelengths in the range 780–1064 nm, but can be transferred back to the GS by irradiation at 660 nm (Fig. 8 ▶), corresponding to an absorption band of the PLI state (Fig. S15 in the supporting information). The second pair, 1858 and 1781 cm^−1^, corresponds to the PLI-2 state. It displays a shift of 95 cm^−1^ for the asymmetric mode and again a comparatively small shift of −8 cm^−1^ for the symmetric mode. These two bands disappear on heating above 50 K (Fig. S11 in the supporting information). Upon this decay, the area of the GS bands at 1866 and 1686 cm^−1^ increases slightly by 8 (2)%, while the other light-induced bands are not affected. Furthermore, these two bands can be erased by illumination at 830 nm (transfer back to GS; see Fig. S12 in the supporting information). The third pair at 1808 and 1775 cm^−1^ corresponds to the PLI-3 state. It displays shifts of 89 and −58 cm^−1^ (or 121 and −92 cm^−1^). It decays at around 90 K (Fig. S13 in the supporting information) and can be erased with IR light in the range 780–1064 nm (Fig. S14 in the supporting information). The population of this PLI-3 state is of the order of 4–5%.

Based on these observations, we can make a tentative assignment of the new bands to possible PLI configurations of the two NO ligands:

(*a*) X-ray diffraction shows the PLI-1 of the bent NO group, with an angle decreasing from 133.88 (9)° (GS) to 109 (1)° (PLI-1) by moving the NO from its original position over the linear NO ligand (O—O = 3.88 Å) to a new position over the Cl ligand (O—Cl = 3.47 Å). IR spectroscopy shows two new bands at 1871 and 1653 cm^−1^ which correspond to this new structure. The down-shift (−33 cm^−1^) of the asymmetric mode could indicate this N-bound PLI-1 configuration of the bent NO group, in agreement with the observed behaviour of {Ru(NO)_2_}^8^-systems (the lower the NO frequency, the lower the *M*—N—O angle). Moreover, we would expect a shift to higher frequencies by about 100 cm^−1^ upon generation of an O-bound PLI, as has been observed in {PtNO}^8^ complexes (Schaniel, Woike *et al.*, 2007 ▶; Schaniel *et al.*, 2009 ▶). The small positive shift (5 cm^−1^) of the symmetric mode can be interpreted as a response (of the linear NO group) to the strong change in electron density by the rotation of the bent NO. In that case it is not necessary to assume a structural change of the linear NO.

(*b*) The second PLI (PLI-2), with its IR signature at 1858 and 1781 cm^−1^, could be assigned to a second PLI of the bent NO ligand, this time with an O-bound configuration. This assignment is based on the observation that the symmetric mode hardly changes while the asymmetric mode shifts to higher wavenumbers, similar to mononitrosyl–platinum compounds (Schaniel, Woike *et al.*, 2007 ▶; Schaniel *et al.*, 2009 ▶), where an already bent NO group switched to an O-bound configuration with a correspondingly increased NO stretching vibration frequency (by about 100 cm^−1^). Since the population is low (about 8%) and the geometry most probably overlaps with the N-bound PLI-1, we are not able to distinguish this configuration in the X-ray diffraction experiment.

(*c*) The third PLI (PLI-3), with its IR signature at 1808 and 1775 cm^−1^, most probably corresponds to a linkage isomer of the linear NO ligand (MS1 or MS2). The significant downshift of the symmetric mode, as well as the fact that it can be erased by IR light, is known from the family of mononitrosyl compounds (Gütlich *et al.*, 2001 ▶).

Overall, this dinitrosyl complex allows for the identification of three PLI configurations, one of which could be determined structurally by X-ray diffraction. It is probable that, by changing ligands and/or counterions, the populations of the different states can be influenced, making possible a large variety of structural configurations.

**Figure 9:**
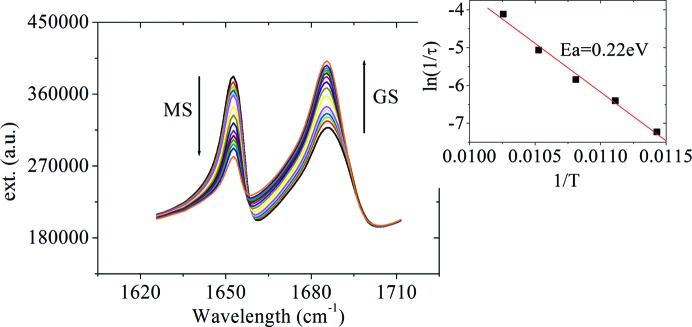
IR spectra of [RuCl(NO)_2_(PPh_3_)_2_]BF_4_ at 87.5 K as a function of time after illumination at 405 nm. From the decrease in the area of the 1653 cm^−1^ band and the increase in the area of the 1686 cm^−1^ band, the corresponding exponential decay curve has been derived and fitted to determine the decay rate constant (*k* = 1/τ) of the PLI-1. Inset: An Arrhenius plot [ln(1/τ) as a function of 1/*T*] to determine the barrier height *E*_a_ between the GS and PLI-1.

Finally, we determined the barrier height between GS and PLI-1 by measuring both the time-dependence of the PLI-1 decay at different temperatures in the range 80–114 K (Fig. 9 ▶) and the decay of the population by recording a spectrum every 2 K. In both cases the decay follows an Arrhenius behaviour. The time-dependence shows a purely exponential decrease and, from the decay of the population, we obtain an isosbestic point between the bands of the GS and PLI-1, as presented in Fig. 9 ▶.

The PLI-1 state decay follows a first-order reaction like all other photo-induced NO isomers, with an activation energy of 0.22 (9) eV (Fig. 9 ▶). This rather low activation energy could be expected from the rather low decay temperature, and is in agreement with observations of the PLI of bent NO groups in the {PtNO}^8^ compounds (Schaniel, Woike *et al.*, 2007 ▶; Schaniel *et al.*, 2009 ▶), where activation energies of 0.27 (3) and 0.18 (3) eV were found.

## 4. Conclusions   

This work addresses several fundamental questions on the possibility of light-induced NO linkage isomerism in dinitrosyl complexes, using [RuCl(NO)_2_(PPh_3_)_2_]BF_4_ of the {Ru(NO)_2_}^8^ type as a prototype. In the title compound, the [RuCl(NO)_2_(PPh_3_)_2_]^+^ cation exhibits a vacant octahedral structure, with one linear nitrosyl ligand in the basal plane of the pyramid and one bent nitrosyl ligand at the apex. On illumination with laser light at 405 nm, several photo-induced linkage isomers (PLIs) have been detected by IR spectroscopy at low temperature, the overall population being 72%. In the IR spectrum for the major contribution to the PLIs (PLI-1), the asymmetrically coupled NO vibrational band shifts by −33 cm^−1^ to lower values, while the symmetrically coupled band splits and shifts by 5 cm^−1^ to higher values and by −8 cm^−1^ to lower values. The down shift is a clear indication of the structural change and the small upward shift in response to the new electronic configuration of the metastable structure. This last has been determined by the photocrystallographic technique at 10 K. The major crystallographic result is that only one of the two nitrosyl ligands, the bent one, undergoes structural changes. In the ground state, the bent nitrosyl is oriented towards the linear equatorial N—O group with an Ru—N—O angle of 133.88 (9)°, and after irradiation at 405 nm the orientation is changed by rotation towards the Cl ligand opposite the linear NO. The Ru—N—O angle in this new position is 109 (1)°. This new orientation is most probably stabilized by two intermolecular hydrogen bonds, with O1⋯H distances of O1⋯H21 = 2.29 (1) and O1⋯H22 = 2.51 (1) Å. On the basis of different structural refinement models, the photocrystallographic analysis provides evidence that, in the photo-induced metastable state PLI-1, the bent NO group is attached to the Ru atom through the N atom (Ru—N—O) rather than in an isonitrosyl Ru—O—N binding mode.

## Supplementary Material

Crystal structure: contains datablock(s) gs. DOI: 10.1107/S2052252514023598/yc5003sup1.cif

Structure factors: contains datablock(s) gs. DOI: 10.1107/S2052252514023598/yc5003gssup2.hkl

Extra figures and tables. DOI: 10.1107/S2052252514023598/yc5003sup3.pdf

CCDC reference: 1036237

## Figures and Tables

**Table 1:** Crystallographic data and refinement details for [RuCl(NO)_2_(PPh_3_)_2_)]BF_4_ in the ground and photo-irradiated states

	Ground state	Photo-irradiated state
*T* (K)	10	10
Formula	RuClN_2_O_2_P_2_C_36_H_30_BF_4_	RuClN_2_O_2_P_2_C_36_H_30_BF_4_
*M*_r_	807.89	807.89
Crystal system	Monoclinic	Monoclinic
Space group	*P*2_1_/*c*	*P*2_1_/*c*
*Z*	4	4
*a* ()	19.0673(6)	19.111(2)
*b* ()	9.8391(2)	9.8741(6)
*c* ()	20.5654(6)	20.588(2)
()	117.580(4)	117.169(14)
*V* (^3^)	3419.7(2)	3456.4(6)
**_calc_ (gcm^3^)	1.560	1.553
(mm^1^)	0.684	0.681
Crystal size (mm)	0.19 0.46 0.48	0.18 0.04 0.04
No. of measured reflections	72353	28875
range ()	2.8733.14	2.8529.57
No. of unique reflections, *R*_int_	13009, 0.0408	8635, 0.0987
No. of reflections with *I*>obs(*I*)†	11370	5511
Refinement program	*SHELXL97* (Sheldrick, 2008 ▶)	*JANA2006* (Petek *et al.*, 2006 ▶)
*N*_var_	442	80
*R*_1_ [*F* ^2^ > obs(*F* ^2^)]† ‡	0.0345 [0.0275]	0.1581 [0.0912]
*wR*_2_ [*F* ^2^ > obs(*F* ^2^)]† §	0.0663 [0.0625]	0.1055 [0.0945]
GoF	1.059¶	1.88††
_max_, _min_ (e^3^)	0.848, 0.840	3.59, 2.80

† obs = 2 for the *SHELXL97* refinement and obs = 3 for the *JANA2006* refinement.

‡ *R*_1_ = |*F*_o_ *F*_c_|/*F*_o_.

§ *wR*_2_ = {[*w*(*F*_o_^2^ *F*_c_^2^)^2^]/[*w*(*F*_o_^2^)^2^]}^1/2^.

¶ Goodness of fit, GoF = {[*w*(*F*_o_^2^ *F*_c_^2^)^2^]/(*N*_obs_ *N*_var_)}^1/2^.

†† GoF = {[*w*(*F*_o_ *F*_c_)^2^]/(*N*_obs_ *N*_var_)}^1/2^.

**Table 2:** Comparison of selected bond distances and angles for the GS and PLI-1 (, )

	GS	PLI-1
RuCl	2.3520(3)	2.331(5)
RuP1	2.4512(4)	2.442(4)
RuP2	2.4436(7)	2.457(4)
RuN1	1.881(1)	2.13(1)
RuN2	1.762(1)	1.729(5)
N1O1	1.164(1)	1.06(3)
N2O2	1.145(1)	1.145(5)
		
RuN1O1	133.88(9)	109(1)
RuN2O2	178.5(1)	175.0(3)
P1RuP2	172.97(1)	171.7(2)
ClRuN1	106.06(4)	109.5(5)
ClRuN2	151.50(1)	158.4(2)
