# Supplementary material for: Multiple light-induced NO linkage isomers in the dinitrosyl complex [RuCl(NO)2(PPh3)2]BF4 unravelled by photocrystallographic and IR analysis
Source: IUCrJ. 2015 Jan 1;2(Pt 1):35–44. doi: 10.1107/S2052252514023598 (PMC4285879; doi:10.1107/S2052252514023598)
Supplement: Supplementary file 3 [file m-02-00035-sup3.pdf]

IUCrJ

Volume 2 (2015)

Supporting information for article:

Multiple light-induced NO linkage isomers in the dinitrosyl complex [RuCl(NO)2(PPh3)2]BF4 unraveled by photocrystallographic and infrared analysis

Nicolas Casaretto, Sebastien Pillet, El Eulmi Bendeif, Dominik Schaniel, Anna K. E. Gallien, Peter Klufers and Theo Woike

Supplementary material of

Multiple light-induced NO linkage isomers in the dinitrosyl complex $[RuCl(NO)_2(PPh_3)_2]BF_4$ unravelled by photocrystallographic and infrared analysis

Structural data

Single crystal diffraction data has been collected at 10K in the ground state (GS) and in the photo-irradiated state after irradiation of the sample at 10K with a diode laser at 405nm (P=70mW) during 40min, until the photo-stationary state is reached.

Ground state structural refinement
Diffraction data were first collected at 10K in the ground state (GS). The corresponding structure was solved in the $P2_1/c$ space group by direct methods with the SHELXS97 program and refined on F^2 by weighted full matrix least-squares methods using the SHELXL97 program. All non-hydrogen atoms were refined anisotropically; hydrogen atoms were located in difference Fourier maps and treated using a riding model, constraining the isotropic displacement parameters to be 1.2 times those of the attached C atom. The refinement converged to agreement factors of R1=0.0345 and wR2=0.0663. The corresponding ORTEP plot is given in figure 1 of the manuscript.

Photo-irradiated state structural refinement
Based on infra-red spectroscopy measurements (*vide supra*), the photo-irradiation conditions should lead to a sufficient conversion ratio. Photo-difference maps (Fig. 2) and Fobs Fourier electron density map (Fig. S3) reveal pronounced structural reorganisation around the bent nitrosyl ligand, with clear indications for the presence of only two molecular species GS and PLI-1. In their neutron diffraction studies of the MS1 and MS2 metastable states of sodium nitroprusside, Schaniel and co-workers measured the transmitted light intensity through the single crystal sample to fix *a priori* the population of metastable state during their structural refinements (Schaniel et al., 2005; Schaniel et al., 2006). On the contrary, in the present work, the population of PLI-1 could not be determined precisely in the diffraction experimental conditions ; the respective population of GS (P_{GS}) and PLI-1 (P_{PLI-1}) are thus necessary parameters of the structural refinement (with the constraint $P_{GS} + P_{PLI-1} = 1$).

The photo-Wilson plot $\log(I_{GS+PLI-1}/I_{GS}) = K-2\Delta B^*(\sin(\theta)/\lambda)^2$ has been calculated (Fig. S4). I_{GS} and $I_{GS+PLI-1}$ are the Bragg intensities of the GS and photo-irradiated state respectively, normalized to a common scale, and ΔB is the GS to photo-irradiated state difference in average atomic displacement parameters. The obtained positive value of the fitted $\Delta B=0.08Å^2$ indicates a systematic increase in atomic displacement parameters in the photo-irradiated structure, resulting from increased thermal vibration effects, or overall disorder generated by the light irradiation. It is unlikely that the increase in ΔB results from an increase in temperature of the sample, since our diffraction data collection was not performed with a

permanent laser irradiation.

Several step by step refinement strategies were applied in order to properly deconvolute the molecular structure of the GS and PLI-1.

Average structural model

In a first step, an average structure was refined starting from the ground state structural model, using similar riding constraints for the hydrogen atoms. The bent nitrosyl group was refined anisotropically assuming a Ru-N1-O1 nitrosyl configuration. In this model, the obtained atomic positions correspond to an average between the ground state and photo-irradiated state positions, weighted by their respective populations P_{GS} and P_{PLI-1}. The corresponding residual electron density map (Fig. S5) clearly show residual features which are not properly described by the model. The ellipsoids of the bent N1-O1 nitrosyl exhibit exaggerated elongated shape, which highlight a heavy atomic disorder. This results from the superposition of two structural configurations for this nitrosyl group. On the contrary, the linear nitrosyl group do not show oversized ellipsoids. At this point, we may suspect that only one nitrosyl group undergoes a light-induced transformation. This is also shown by the refined values of the atomic displacement parameters given in table S1.

Average + 2 nitrosyl N1-O1 model

In a second step, two initial configurations were introduced for the bent NO1 nitrosyl group based on the positions of the diffraction peaks in the calculated photo-difference map (Fig. 2), one corresponding to the GS molecular species with Ru-N1A-O1A nitrosyl configuration, the other one corresponding to the PLI-1 species, assuming a Ru-N1B-O1B nitrosyl configuration. The two groups were refined separately and isotropically using N-O distance restraints to 1.16Å. For the least-squares refinement, a random spatial distribution of the PLI-1 species was considered with the formalism

$F_{photo\text{-}irradiated}(hkl) = P_{GS}*F_{GS}(hkl) + P_{PLI}*F_{PLI\text{-}1}(hkl)$ with the constraint $P_{GS} + P_{PLI\text{-}1} = 1$ (Vorontsov & Coppens, 2005).

The corresponding agreement factors R1 and wR2 decrease with respect to the Average model, while the residual electron density (Fig. S6) is much more featureless. The refined atomic displacement parameters for the N1-O1 and N2-O2 are in the same range, which indicates that the proposed structural model is much more appropriate. The refined population of PLI-1 is 42.1(5).

GS-rigid group + PLI-1-rigid group + nitrosyl N1-O1 model

In a third model, the complete molecular structure of the ground state, corrected for the change of lattice parameters on irradiation, was introduced and refined as a rigid group (rigid group 1). The atomic displacement parameters are fixed at the GS structure values for all the atoms but the O1A and N1A atoms of the bent nitrosyl, which are refined anisotropically. For the PLI-1 species, the Ru and the O1B and N1B atoms of the bent nitrosyl are freely refined anisotropically in a Ru-N1B-O1B nitrosyl configuration. The remaining atoms of the PLI molecule are treated as a rigid group (rigid group 2) using the GS structure. The atomic displacement parameters of the PLI-1 N1B and O1B were constrained to have the same values than the refined ones of the GS N1A and O1A. The refined population of PLI-1 is 52(1)%. The agreement statistic factors for this model are R1 = 0.1581, and wR2 = 0.1055. The refinement of the PLI-1 as a nitrosyl (Ru-N1B-O1B case) results in quite consistent atomic displacement parameters for both N1B (Uiso(N1B) = 0.028(4) $Å^2$) and O1B (Uiso(O1B) = 0.029(4) $Å^2$). This model is much more constrained than the previous one, but the number of refined parameters is reduced drastically (from 439 to 80!). The structural degrees of freedom

we introduced, refining the position and thermal parameters of N1, O1, and Ru, allow taking care of the reorganisation in the photo-induced state of the bent nitrosyl and displacement of the Ru atom from the basal plane containing N2, O2, P1, P2, and Cl.

GS-rigid group + PLI-1-rigid group + isonitrosyl O1-N1 model

In a fourth model, the hypothesis for an isonitrosyl configuration of PLI-1 was tested, using the same strategy than for the previous model (*GS-rigid group + PLI-1-rigid group + nitrosyl N1-O1 model*). The results of the structural refinement are very similar, with a refined population of PLI-1 of 55(1)%, and agreement statistic factors of R1 = 0.1577, and wR2 = 0.1053, marginally lower than for the nitrosyl configuration. The refinement of the PLI-1 as an isonitrosyl (Ru-O1B-N1B case), leads to unreasonably high values for O1B (Uiso(O1B) = 0.041(4) $Å^2$, Uiso(N1B) = 0.018(4) $Å^2$). This is an indication that the assignment of the atom connected to Ru (O1B) as an oxygen is probably wrong, and must rather be taken as a nitrogen.

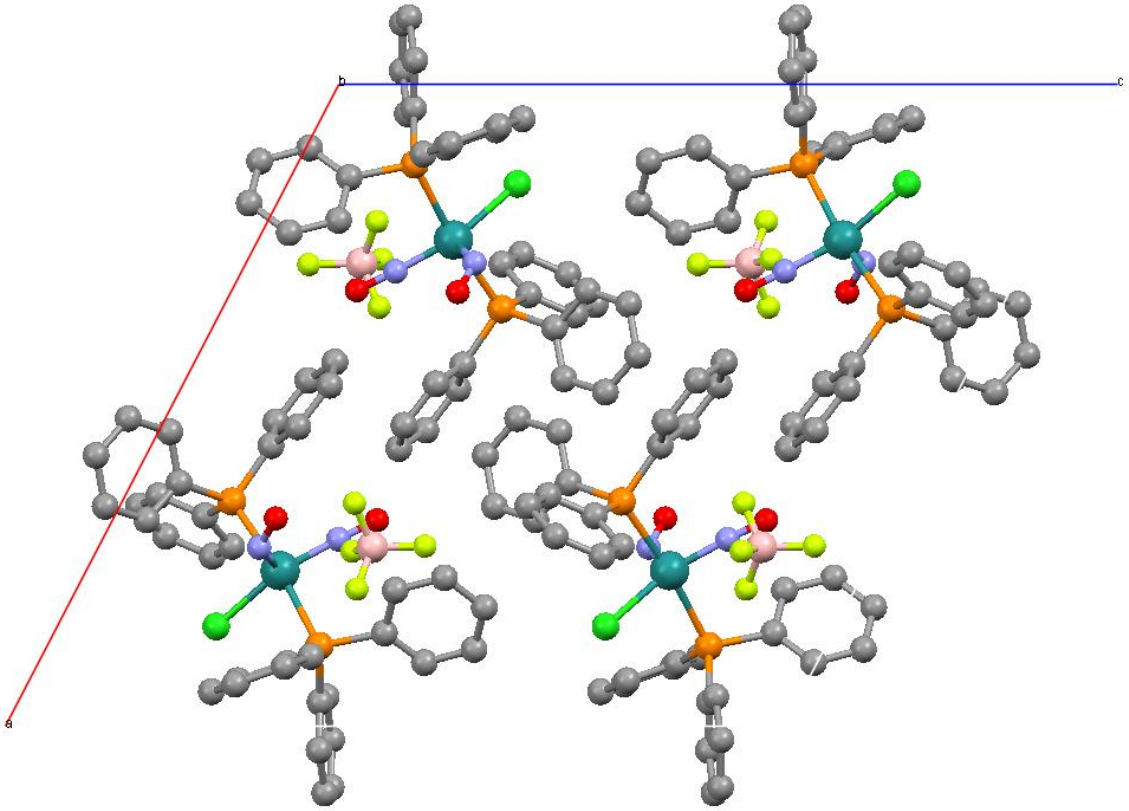

Fig. S1 Packing diagram of $[RuCl(NO)_2(PPh_3)_2]BF_4$ in the GS viewed along the *b* axis.

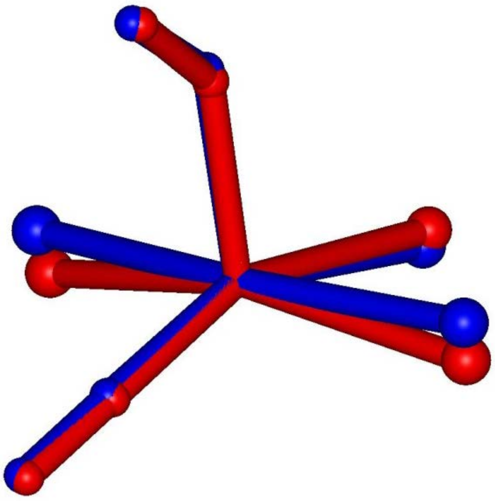

Fig. S2. Superposition of the coordination geometry of the $[RuCl(NO)_2(PPh_3)_2]BF_4$ (in blue) and $[RuCl(NO)_2(PPh_3)_2]PF_6$ (in red) analogues.

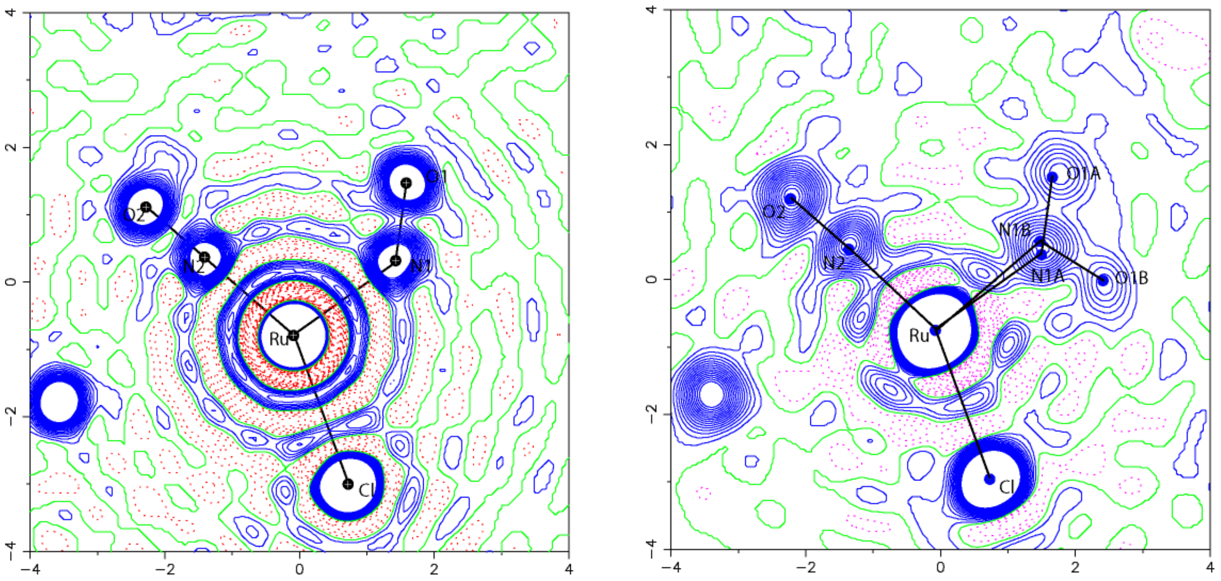

Fig. S3. Fobs Fourier electron density map calculated in the Ru-N1-N2 plane, with contours of ±1.00eÅ^{-3} (red, negative; blue, positive) for the (left) ground state, and (right) PLI-1 state.

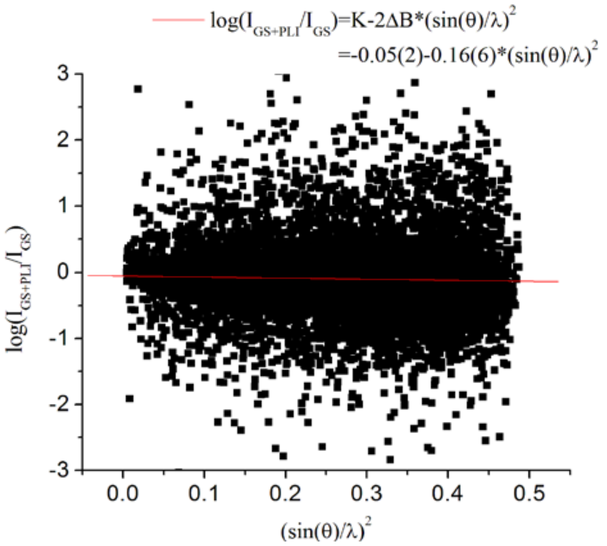

Fig. S4 Photo-Wilson plot in the form of $\log(I_{GS+PLI-1}/I_{GS})$ as a function of $(\sin(\theta)/\lambda)^2$, where the Bragg intensities of the GS I_{GS} and photo-irradiated state $I_{GS+PLI-1}$ have been normalized to a common scale using SHELXL. ΔB is the GS to photo-irradiated state difference in average atomic displacement parameters.

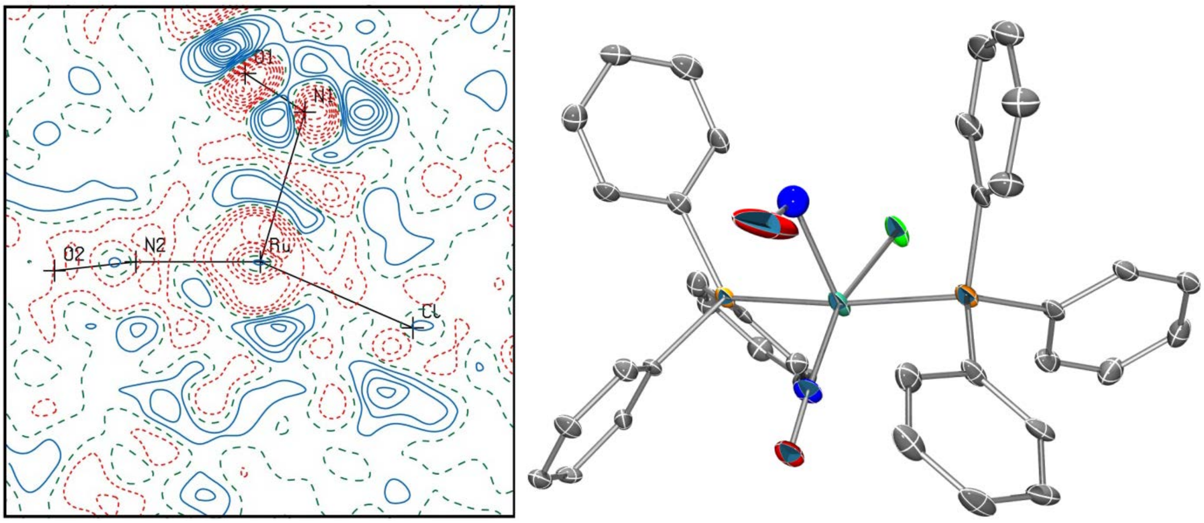

Fig. S5 Left: Residual electron density map calculated in the Ru-N1-N2 plane, with contours of ±0.25eÅ^{-3} (red, negative; blue, positive) for the Average refinement strategy. Right : Ortep view, ellipsoids are plotted at the 50% probability level.

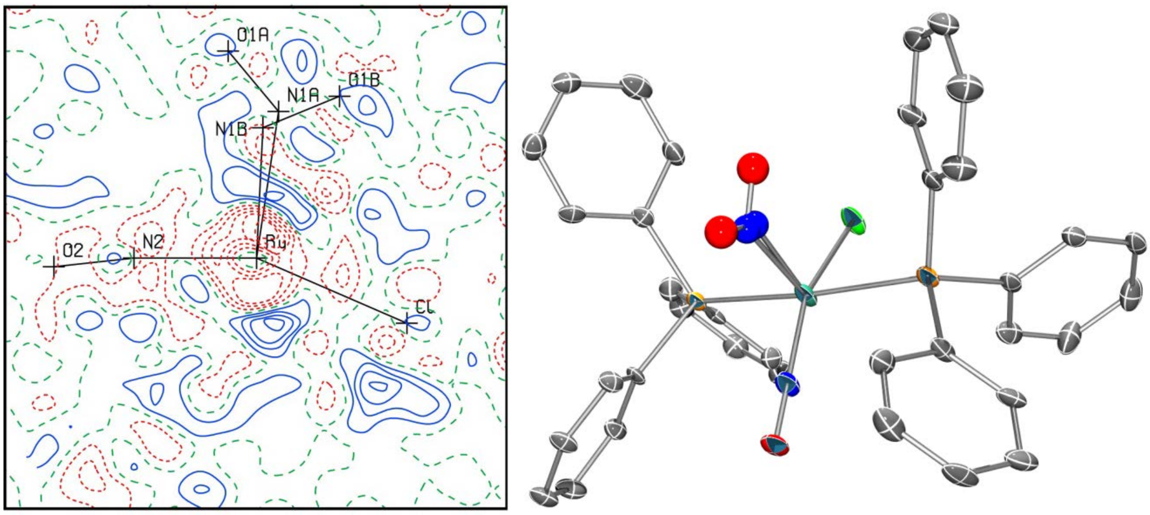

Fig. S6 Left: Residual electron density map calculated in the Ru-N1-N2 plane, with contours of ±0.25eÅ^{-3} (red, negative; blue, positive) for the Average + 2 N-O refinement strategy. Right : Ortep view, ellipsoids are plotted at the 50% probability level.

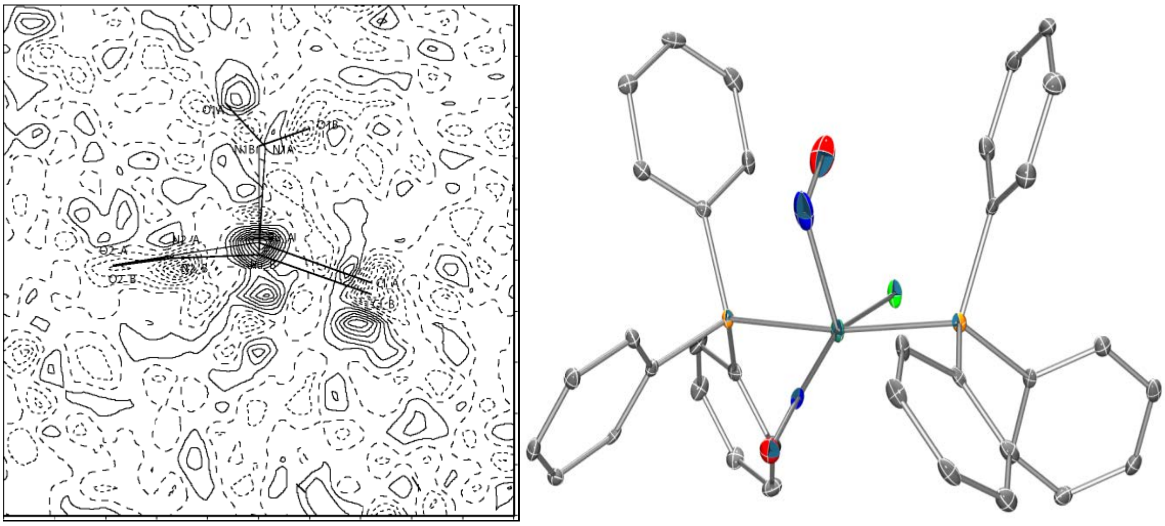

Fig. S7 Left: Residual electron density map calculated in the Ru-N1-N2 plane, with contours of ±0.25eÅ^{-3} (dotted, negative; continuous, positive) for the GS-rigid group + PLI-1-rigid group + nitrosyl N1-O1 refinement strategy. Right: ORTEP view of the PLI molecule only (the GS molecule has been omitted for clarity), ellipsoids are plotted at the 50% probability level.

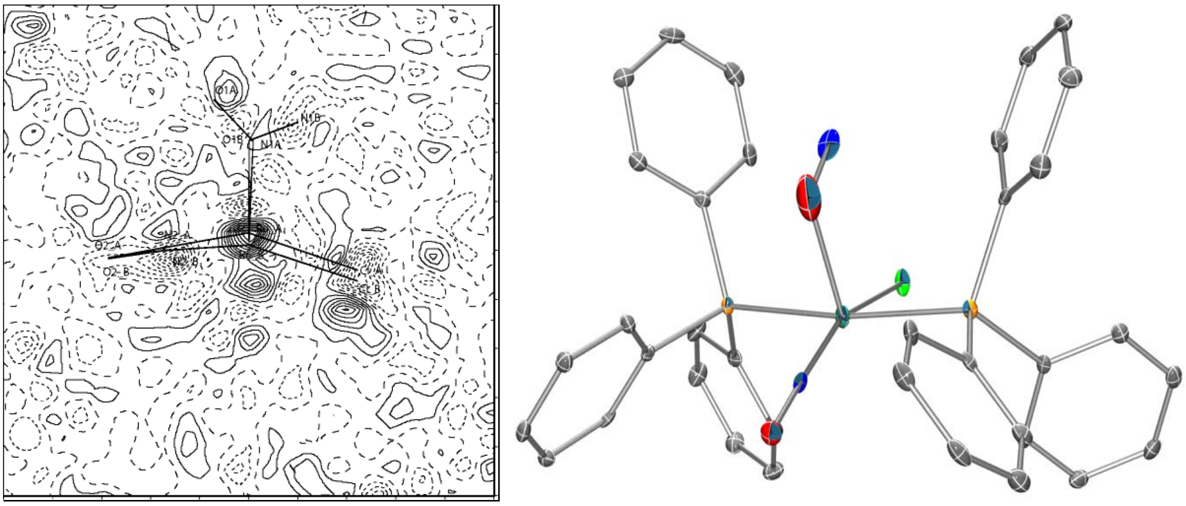

Fig. S8 Left: Residual electron density map calculated in the Ru-N1-N2 plane, with contours of ±0.25eÅ^{-3} (red, negative; blue, positive) for the GS-rigid group + PLI-1-rigid group + isonitrosyl O1-N1 refinement strategy. Right: Ortep view oft he PLI-1 molecule only (the GS molecule has been omitted for clarity), ellipsoids are plotted at the 50% probability level.

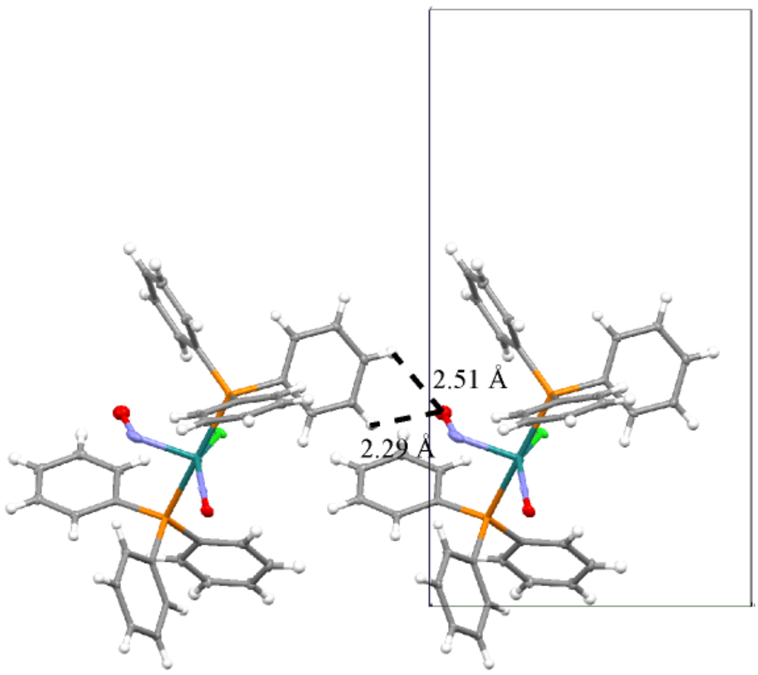

Fig. S9. Intermolecular contacts involving the bent nitrosyl of $[RuCl(NO)_2(PPh_3)_2]BF_4$ in the PLI-1.

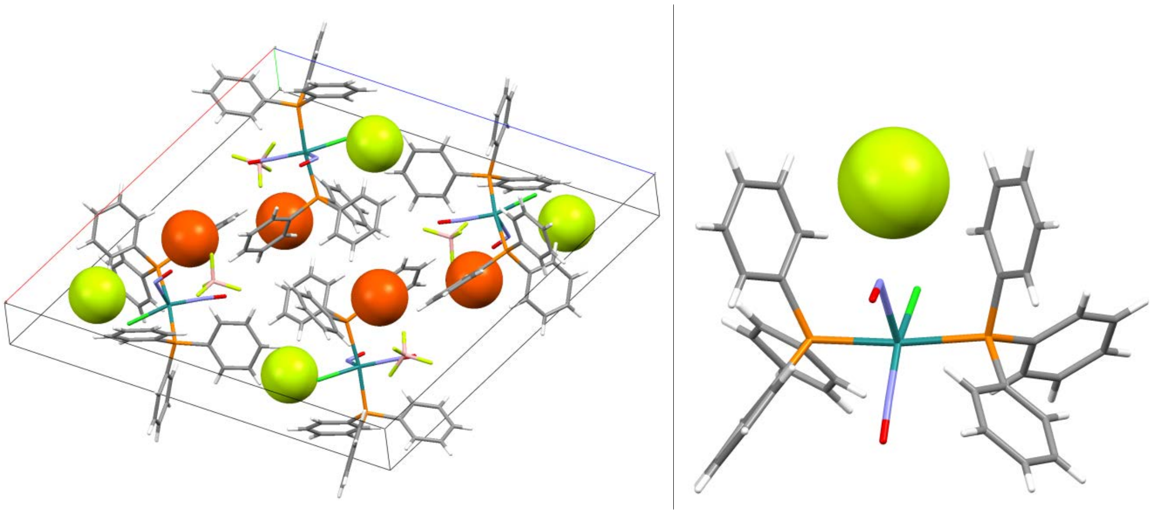

Fig. S10. Location of the free cavity in the crystal packing. The green-yellow sphere corresponds to a cavity near to the position of the bent NO group after photo-isomerisation to the PLI-1 state.

Infrared-spectroscopy data

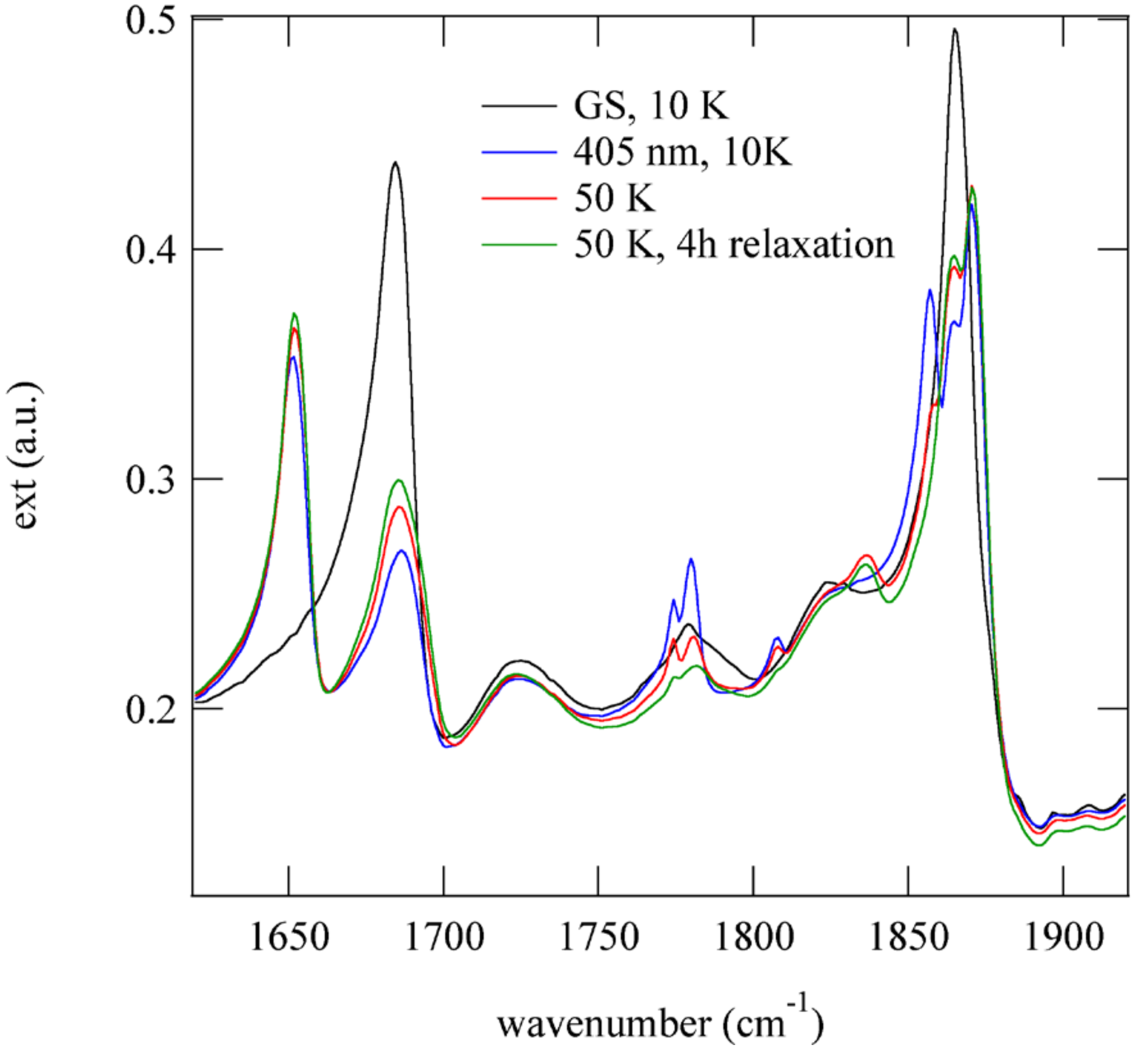

Fig. S11. Thermal depopulation of PLI-2: Infrared spectra of $[RuCl(NO)_2(PPh_3)_2]BF_4$ at 10K in GS (black line) and after illumination at 405 nm to populate the PLIs (blue line). Disappearing of the 1858 cm^{-1} and 1781 cm^{-1} bands when heating above 50 K.

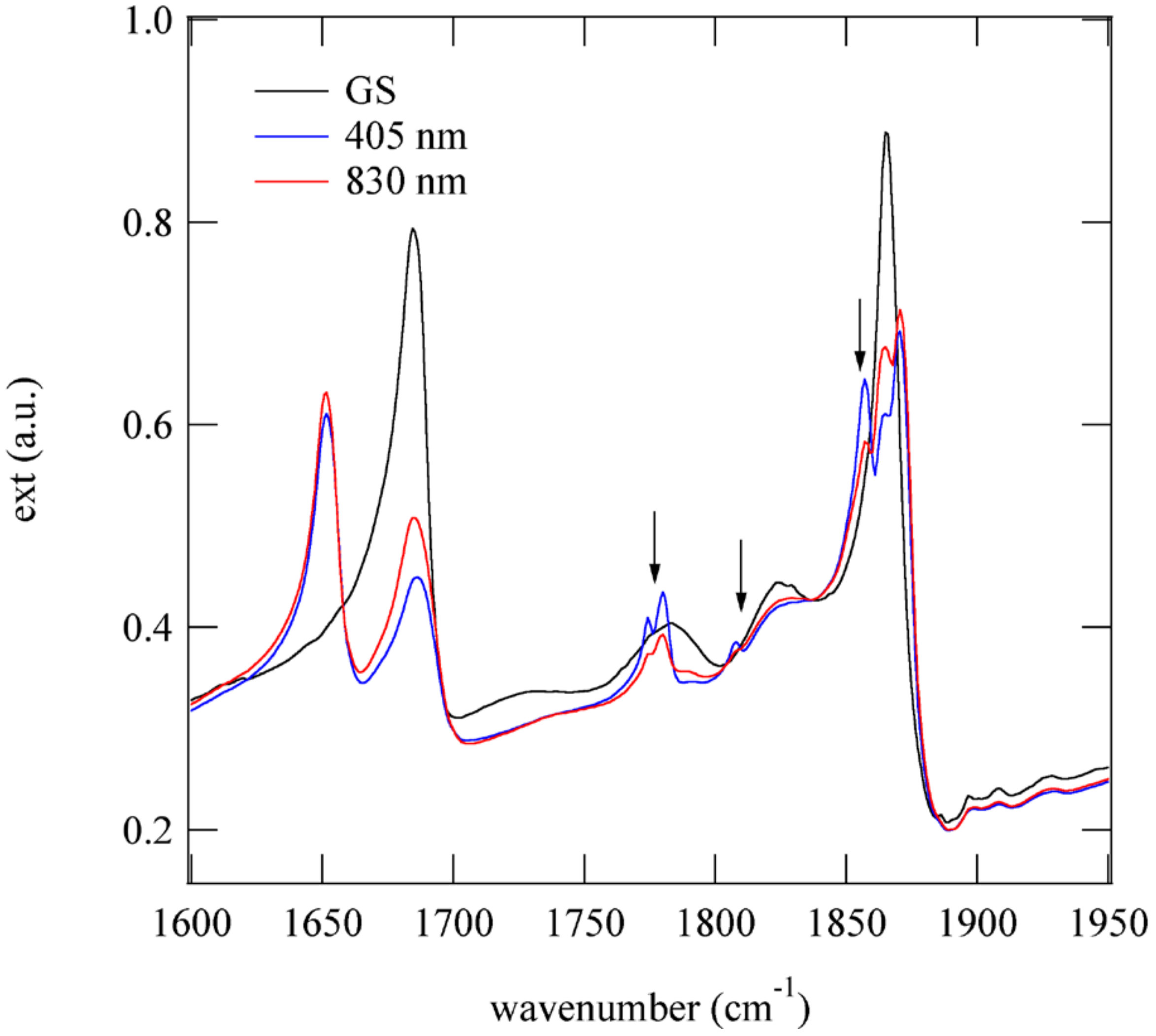

Fig. S12. Light-induced depopulation of PLI-2: Infrared spectra of $[RuCl(NO)_2(PPh_3)_2]BF_4$ at 10K in GS (black line) and after illumination at 405 nm to populate the PLIs (blue line). Back transfer of the 1858 cm^{-1} and 1781 cm^{-1} bands by illumination at 830 nm.

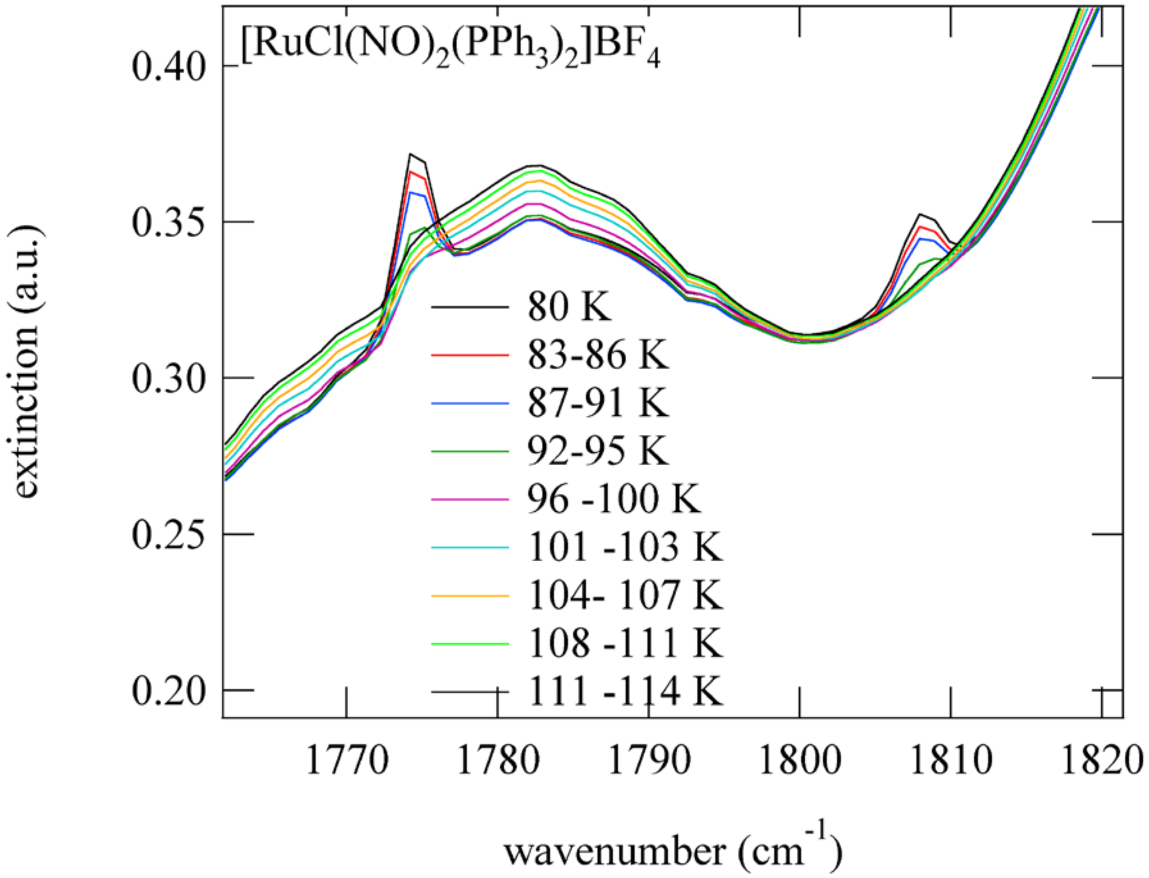

Fig. S13. Thermal depopulation of PLI-3: Decay of the two bands at 1808 cm^{-1} and 1775 cm^{-1} at around 90K.

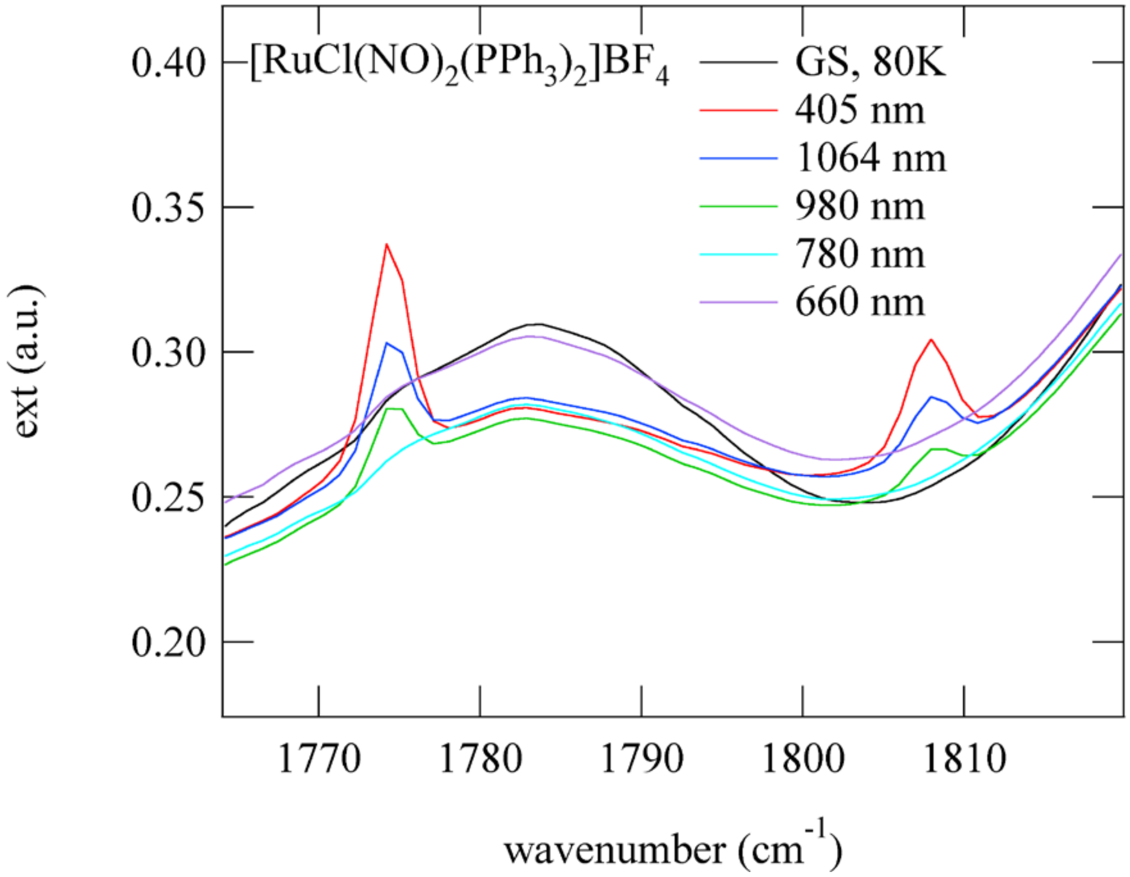

Fig. S14. Light-induced depopulation of PLI-3: Infrared spectra of $[RuCl(NO)_2(PPh_3)_2]BF_4$ at 80K in GS (black line) and after illumination at 405 nm to populate the PLIs (red line). Back transfer of the 1808 cm^{-1} and 1775 cm^{-1} bands by illumination with infrared light in the range 780-1064 nm.

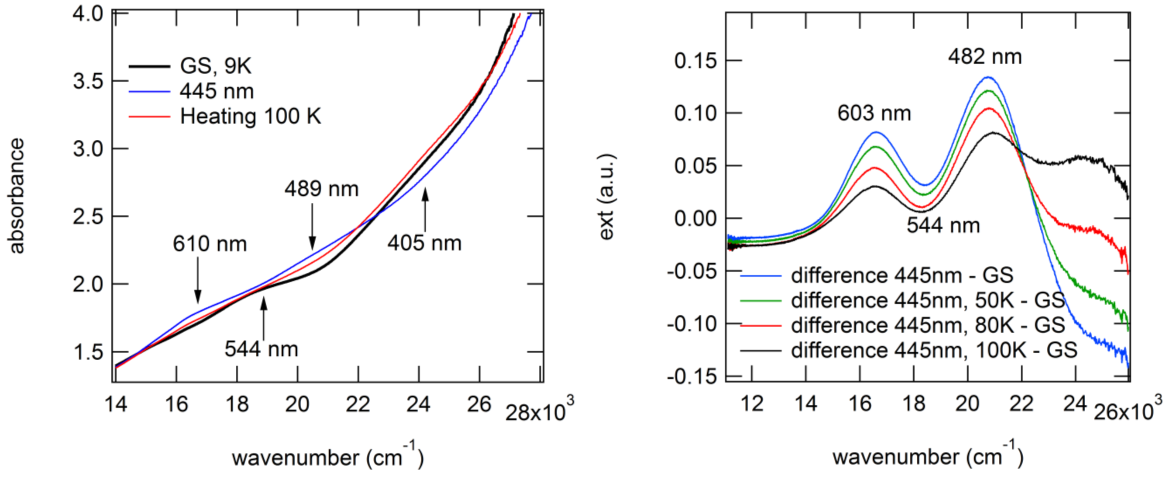

Fig. S15. Left: UV-vis absorption spectra in transmission through a KBr pellet in the GS, after photo-excitation at 445nm, and subsequent warming to 100K. Right: difference spectra.

Table S1. Refinement details for $[RuCl(NO)_2(PPh_3)_2)]BF_4$ (**1**) in the ground state (GS) and photo-irradiated state (GS+PLI-1).

	Ground state	Photo-irradiated			
Refinement strategy		Average	Average + 2 nitrosyl N1-O1	GS-rigid group + PLI-1-rigid group + nitrosyl N1-O1	GS-rigid group + PLI-1-rigid group + isonitrosyl O1-N1
No. of variables	442	442	439	80	80
No. of constraints					
Refinement program	SHELXL	SHELXL	SHELXL	JANA2006	JANA2006
[a,d]R1 [F^2 > obs*s(F^2)]	0.0816 [0.0522]	0.1238 [0.0728]	0.1174 [0.0664]	0.1581 [0.0912]	0.1577 [0.0910]
[b,d]wR2 [F^2 > obs*s(F^2)]	0.1174 [0.1013]	0.1707 [0.1404]	0.1485 [0.1213]	0.1055 [0.0945]	0.1053 [0.0943]
GoF	[c]1.039	[c]1.043	[c]1.039	[e]1.88	[e]1.88
$\Delta\rho_{max,\ min}$ (eÅ^{-3})	2.542 / -1.251	1.919 / -1.821	1.310 / -0.989	3.59 / -2.80	3.71 / -2.83
Refined population of PLI (P_{PLI})	/	/	42.1(5)%	52(1)%	55(1)%
Ueq(Ru)	0.00820(7)	0.01812(14)	0.0180(1)	0.0107(3)	0.0107(3)
Ueq(N2)<br>Ueq(O2)	0.0083(5)<br>0.0145(5)	0.021(1)<br>0.0258(9)	0.0211(9)<br>0.0265(8)	0.0083<br>0.0145	0.0083<br>0.0145
Ueq(N1A)<br>Ueq(O1A)	0.0134(6)<br>0.0279(6)	0.135(6)<br>0.178(6)	0.0206(13)<br>0.0324(12)	0.028(4)<br>0.029(4)	0.018(4)<br>0.041(4)
Ueq(N1B)<br>Ueq(O1B)	/	/	0.0206(13)<br>0.0324(12)	0.028(4)<br>0.029(4)	0.041(4)<br>0.018(4)

[a]R1 = $\Sigma|Fo\text{-}Fc|/Fo$. [b]wR2 = $\{\Sigma[w(F_o^2-F_c^2)^2]/\ \Sigma[w(F_o^2)^2]\}^{1/2}$. [c]GoF = $\{\Sigma[w(F_o^2-F_c^2)^2]/\ (N_{obs}-N_{var})\}^{1/2}$. [d]obs = 2 for the SHELXL refinement and obs = 3 for the JANA2006 refinement. [e]GoF = $\{\Sigma[w(F_o-F_c)^2]/\ (N_{obs}-N_{var})\}^{1/2}$.

References

Schaniel, D., Woike, T., Schefer & J., Petříček, V. (2005). *Phys. Rev*. B **71**, 174112.

Schaniel, D., Woike, T., Schefer, J., Petříček, V., Krämer, K. W. & Güdel, H. U. (2006). *Phys. Rev*. B **73**, 174108.

Vorontsov, I. I. & Coppens, P. (2005). *J. Synchrotron Rad.* **12**, 488-493.
